# Postoperative Patient Pain Severity and Its Association With Anxiety, Depression, and Sleep Quality

**DOI:** 10.7759/cureus.54553

**Published:** 2024-02-20

**Authors:** Mariwan Husni, Haitham Jahrami, Hamdi Al Shenawi, Saleh F Alenenzi, Faisal N Alhawas, Mohammed A Asiri, Fayza Haider, Ahmad F Alanazi, Rami J Yaghan

**Affiliations:** 1 Psychiatry, Arabian Gulf University, Manama, BHR; 2 Psychiatry, Northern Ontario School of Medicine University, Thunder Bay, CAN; 3 Surgery, Arabian Gulf University, Manama, BHR; 4 Pediatric Surgery, Salmaniya Medical Complex, Manama, BHR; 5 Psychiatry, University of Dammam, Dammam, SAU; 6 Surgery and Urology, Faculty of Medicine, Jordan University of Science and Technology, Irbid, JOR

**Keywords:** postoperative anxiety, postoperative depression, postoperative pain, pain progression, postsurgical depression, postsurgical anxiety, postsurgical insomnia, psychogenic pain, postsurgical pain

## Abstract

Introduction

The experience of pain is a complex phenomenon. A patient in the acute postsurgical pain setting may feel a constant bombardment of nociceptive input from the surgical site; this in turn influences psychological factors that determine the overall emotional experience of pain, which is significant. The aim of our study was to investigate the severity of pain in postsurgical patients three days after surgery using the 100 mm visual analog scale (VAS).

Methods

This was a cross-sectional assessment of postoperative pain. Participants were patients between 18 and 64 years of age who had undergone a surgical procedure (laparoscopic or open surgery), three days prior to the data collection and who were admitted or discharged postoperatively at the Al Salmaniya Complex, Manama, Bahrain. Participants were asked demographic questions about whether they had laparoscopic or open surgeries and completed self-reporting scales. Patient Health Questionnaire-9 (PHQ-9) was utilized to screen for both the presence and severity of depression; Generalized Anxiety Disorder 7-item (GAD-7) was administered to screen for anxiety; the Insomnia Severity Index (ISI) was used to evaluate insomnia; and the VAS was used to evaluate pain.

Results

Sixty-seven patients were recruited, with a mean age of 61.53 years (SD = 7.37). Twenty-nine (43%) were females, 38 (57%) were males, 36 (54%) underwent elective surgery, 31 (46%) underwent emergency surgery, 31 (46%) underwent laparoscopic surgery, and 36 (54%) underwent open surgery. The average score on the Brief Pain Inventory Short Form (BPISF) was 8.12 (SD = 1.16), indicating a moderate level of pain. Twenty-six (43%) patients had moderate-severe insomnia, 21 participants (31%) had no insomnia, 17 participants (25%) had subthreshold insomnia, 28 (42%) had moderate depression, five (7%) had moderate-severe depression, and 34 (51%) had severe depression. Eighteen participants (27%) had mild anxiety, 46 (69%) had moderate anxiety, and 3 (4%) had severe anxiety. Six of the participants (9%) reported moderate pain, while 61 participants (91%) reported severe pain.

## Introduction

According to the International Association for the Study of Pain (IASP), pain is defined as a negative sensory and emotional experience that occurs when there is actual or potential damage to tissues or when it is described in terms of such damage [1]. The experience of pain is a complex phenomenon. Initially, nociception occurs, which is the ‘physiological’ component of pain; subsequently, experiential patient suffering occurs, which is the ‘emotional subjective’ component [2]. The perceptual and affective experience of pain is influenced not only by the amount of the bodily lesion alone but also by the personality of the person [3].

A patient in the acute postsurgical pain setting may feel a constant bombardment of nociceptive input from the surgical site; this in turn influences psychological factors that determine the overall emotional experience of pain, which is significant [4].

In the breast cancer population, patients undergoing radical modified mastectomy, anxiety, depression, and reported to be unmarried correlated with greater postoperative pain and dissatisfaction with postoperative analgesia and patient satisfaction [5].

Commonly, physiological disorders and comorbidities are routinely identified and managed preoperatively. However, assessing and managing psychological conditions, as a routine part of the preparation of a patient before surgery, can have an additive positive outcome [6]. This approach may have short-term and long-term benefits, such as reducing progression to chronic postoperative pain syndrome [7].

Many clinical studies have reported that the ‘anxiety’ status of a patient may be firmly associated with the intensity of the surgical pain experience. Higher preoperative anxiety scores increase pain intensity [8]. In numerous clinical studies, it has been consistently observed that there is a strong correlation between a patient's anxiety levels before undergoing surgery and the intensity of their pain experience during and after the procedure. These studies have found that, when individuals have higher scores, indicating greater anxiety prior to surgery, they tend to experience more intense levels of pain. In other words, heightened preoperative anxiety is associated with an increased perception of pain intensity [7-9].

There are a limited number of studies exploring pain care in Middle Eastern or Gulf Cooperation Council (GCC) countries [9]. To the best of our knowledge, this is the first study analyzing and evaluating psychological factors influencing acute postsurgical pain and its progression.

The purpose of this study was to investigate the severity of pain in postsurgical patients using the 100 mm visual analog scale (VAS) three days postoperatively (following open or laparoscopic procedures) and to evaluate the associations of pain severity with anxiety, depression, and sleep severity scores using the Generalized Anxiety Disorder 7-item (GAD-7), Patient Health Questionnaire-9 (PHQ-9), and Insomnia Severity Index (ISI). The 100 mm VAS score is commonly used to measure pain intensity after surgery. However, the minimal change in the VAS score indicates that there is a relevant change in a patient’s pain status [10]. The VAS has been widely adopted as a standard tool for pain assessment due to its simplicity and ease of use. It allows patients to provide a self-reported rating of their pain, providing valuable information for healthcare professionals in evaluating the effectiveness of pain management interventions [10]. 

Identifying any psychological factors during the subacute postoperative period and appropriately managing them may halt the progression to chronic postsurgical pain (CPSP) in relation to the prevalence of psychological problems, including generalized anxiety, depression, impulsivity, and borderline personality disorder, by using validated measures in postsurgical patients in Bahrain.

## Materials and methods

Ethics approval

This study was approved by the Research Ethics Committee (REC), Arabian Gulf University, College of Medicine and Medical Sciences. The approval number is E031-PI-9/21.

Our study was a cross-sectional assessment of postoperative pain (between the third and seventh postoperative days) among patients who underwent surgery at the Salmaniya Medical Complex, Manama, Bahrain. All the scales were in the Arabic language. A written informed consent was attained from all participants. We did not keep any specific information that could have revealed any specific participant identification.

Patients who underwent either open or laparoscopic general surgery were recruited. Age, sex, marital status, education, method, and length of indication for surgery were among the demographic data gathered.

The inclusion criteria for patients were as follows: fluency in the Arabic language (to minimize the influence of culture on the psychiatric diagnosis), had undergone a surgical procedure (laparoscopic or open surgery) three days prior to the data collection, and were admitted or discharged postoperatively at the Salmaniya Medical Complex. Participants were between the ages of 18 and 64 years (to exclude the possibility of chronic illness onset).

The exclusion criteria were as follows: not fluent in the Arabic language; had any history of chronic pain that was not related to the current surgical condition warranting the operation; had any established diagnosis of concurrent mental or chronic illnesses (such as diabetes and hypertension); had any history of addiction-related problems; and had a current history of dependence/abuse on psychiatric medications, hypnotics, painkillers, or tranquilizers.

All participants were asked to complete the following self-reporting scales: PHQ-9 to screen for both the presence and severity of depression [11], GAD-7 to screen for anxiety [12], ISI for evaluation of insomnia [13], and Brief Pain Inventory Short Form (BPISF) [14].

Statistics

Sample Size Calculation

For this cross-sectional study, the sample size was determined by selecting 40% of the available patients over the past six months of the study period as a convenient sample.

Data Analysis

Descriptive statistics were utilized to outline participants' sociodemographic characteristics. The data collected were tested formally for normality. Both the mean and standard deviation (SD) are reported for the continuous variables, and the percentage and count are reported for the categorical variables.

Correlation analysis, in the form of Pearson's product-moment correlation coefficient, calculates the strength of the linear correlation between the chosen variables in the study. A p value of < 0.05 was used to indicate statistical significance. All analyses were performed using Stata software (version 15.0; StataCorp LLC, College Station, Texas).

## Results

The study participants had a mean age of 61.53 years (SD = 7.37). The average ISI score was 13.63 (SD = 8.07), indicating a moderate level of insomnia symptoms. On PHQ-9, participants had an average score of 20 (SD = 4.28), suggesting a high level of depressive symptoms. The GAD-7 scale showed an average score of 11.39 (SD = 2.15), indicating moderate anxiety symptoms. The average score on the BPISF was 8.12 (SD = 1.16), representing a moderate level of pain. In terms of demographic characteristics, 29 participants (43%) identified as female, while 38 participants (57%) identified as male. Regarding the surgery schedule, 36 participants (54%) had elective surgeries, while 31 participants (46%) had emergency surgeries. The surgical approach varied, with laparoscopic surgeries accounting for 31 cases (46%) and open surgeries for 36 cases (54%). Among the participants, 33 individuals (49%) reported no family history of mental illness, while 34 individuals (51%) reported having a family history of mental illness. In terms of education, 17 participants (25%) had a college degree, 15 participants (22%) had completed elementary education, 10 participants (15%) had no formal education, and 25 participants (37%) had completed secondary education. Regarding sleep disturbances, 21 participants (31%) reported no insomnia, 17 participants (25%) had subthreshold insomnia, 16 participants (24%) experienced moderate insomnia, and 13 participants (19%) reported severe insomnia symptoms. In terms of depression severity, 28 participants (42%) had moderate depression, five participants (7%) had moderate to severe depression, and 34 participants (51%) had severe depression. The prevalence of anxiety disorders showed that 18 participants (27%) had mild anxiety, 46 participants (69%) had moderate anxiety, and three participants (4%) had severe anxiety. Finally, the majority of participants reported experiencing severe pain, with 61 individuals (91%) indicating severe pain levels, while only six participants (9%) reported moderate pain levels (Table 1).

**Table 1:** The results of the study variables (n = 67) Data are expressed as mean ± standard deviation (SD) or frequency counts and corresponding percentages.

Continuous Variables	Mean	SD
Age	61.53	7.37
ISI	13.63	8.07
PHQ-9	20	4.28
GAD-7	11.39	2.15
BPISF	8.12	1.16
Categorical variables	Count	Percent
Sex		
Female	29	43%
Male	38	57%
Surgery schedule		
Elective	36	54%
Emergency	31	46%
Surgery approach		
Laparoscopic	31	46%
Open	36	54%
Family history of mental illness		
No	33	49%
Yes	34	51%
Education		
College	17	25%
Elementary	15	22%
None	10	15%
Secondary	25	37%
Insomnia		
No insomnia	21	31%
Subthreshold insomnia	17	25%
Moderate insomnia	16	24%
Severe insomnia	13	19%
Depression		
Mild depression	Nil	Nil
Moderate depression	28	42%
Moderate to severe depression	5	7%
Severe depression	34	51%
Anxiety		
Mild anxiety	18	27%
Moderate anxiety	46	69%
Severe anxiety	3	4%
Pain		
Moderate	6	9%
Severe	61	91%

The correlation analysis revealed several relationships among the variables. The ISI showed a weak positive correlation with the PHQ-9 scores, with Pearson's r correlation coefficient of 0.19 (p = 0.131, n = 67). However, this correlation was not statistically significant. Similarly, the correlation between ISI and the GAD-7 scores was weak, with Pearson's r of 0.16 (p = 0.207, n = 67), indicating no significant association. When examining the relationship between the PHQ-9 and GAD-7 scores, a weak positive correlation of 0.07 was observed (p = 0.586, n = 67). Although the correlation was positive, it was not statistically significant. In terms of the relationship between the ISI and the BPISF scores, a negligible correlation of 0.02 was found (p = 0.866, n = 67), indicating no significant association between the variables. Furthermore, the correlation between the PHQ-9 and BPISF scores revealed a weak positive relationship, with Pearson's r of 0.18 (p = 0.152, n = 67). However, this correlation was not statistically significant. Lastly, the correlation between the GAD-7 and BPISF scores was also weak, with Pearson's r of 0.09 (p = 0.466, n = 67), indicating no significant association between anxiety symptoms and pain levels. The results of the correlation matrix indicating the relationships between the ISI, PHQ-9, GAD-7, and BPISF are summarized in Table 2.

**Table 2:** Correlation analysis of the study variables (ISI, PHQ9, GAD7, and BPISF) (n = 67) Data are expressed as Pearson-product correlation r.

		ISI	PHQ-9	GAD-7	BPISF
ISI	Pearson's r	—			
	Df	—			
	p value	—			
	N	—			
PHQ-9	Pearson's r	0.19	—		
	Df	65	—		
	p value	0.131	—		
	N	67	—		
GAD-7	Pearson's r	0.16	0.07	—	
	Df	65	65	—	
	p value	0.207	0.586	—	
	N	67	67	—	
BPISF	Pearson's r	0.02	0.18	0.09	—
	Df	65	65	65	—
	p value	0.866	0.152	0.466	—
	N	67	67	67	—

An independent sample t-test was conducted to compare the means of different groups for each variable. For the ISI, the t-test yielded a non-significant result (t(65) = -0.68, p = 0.502), indicating no significant difference between the female (M = 12.86, SD = 7.27) and male (M = 14.21, SD = 8.67) groups. The effect size, as measured by Cohen's d, was -0.17. Similarly, for PHQ-9, the t-test revealed no significant difference between the female (M = 19.97, SD = 4.75) and male (M = 20.03, SD = 3.95) groups (t(65) = -0.06, p = 0.955, Cohen's d = -0.01). Regarding GAD-7, the t-test indicated no significant difference between the female (M = 11.69, SD = 2.30) and male (M = 11.16, SD = 2.02) groups (t(65) = 1.01, p = 0.319, Cohen's d = 0.25). Additionally, for the BPISF, the t-test showed no significant difference between the female (M = 8.00, SD = 1.28) and male (M = 8.21, SD = 1.07) groups (t(65) = -0.73, p = 0.467, Cohen's d = -0.18). The results of the independent samples t-test between male and female participants for the ISI, PHQ-9, GAD-7, and BPISF are summarized in Table 3.

**Table 3:** Independent sample t-test between male and female participants for the ISI, PHQ-9, GAD-7, and BPISF A p value of less than 0.05 is considered statistically significant

Variable	Group	N	Mean	SD	p value
ISI	Female	29	12.86	7.27	0.502
	Male	38	14.21	8.67	
PHQ-9	Female	29	19.97	4.75	0.955
	Male	38	20.03	3.95	
GAD-7	Female	29	11.69	2.3	0.319
	Male	38	11.16	2.02	
BPISF	Female	29	8	1.28	0.467
	Male	38	8.21	1.07	

For the ISI, the t-test yielded a non-significant result (t(65) = -0.90, p = 0.373), with a small effect size (Cohen's d = -0.22). Similarly, for PHQ-9, the t-test also showed no significant difference between the groups (t(65) = 0.63, p = 0.533), with a small effect size (Cohen's d = 0.15). However, for GAD-7, the t-test revealed a significant difference between the groups (t(65) = 2.49, p = 0.015), with a moderate effect size (Cohen's d = 0.61). Regarding the BPISF, the t-test did not find a significant difference between the groups (t(65) = -1.12, p = 0.267), with a small effect size (Cohen's d = -0.27).

Table 4 presents the independent sample t-test between elective and emergency participants for the ISI, PHQ-9, GAD-7, and BPISF.

**Table 4:** Independent sample t-test between elective and emergency participants for the ISI, PHQ-9, GAD-7, and BPISF A p value of less than 0.05 is considered statistically significant.

Variable	Group	N	Mean	SD	p value
ISI	Elective	36	12.81	7.88	0.373
	Emergency	31	14.58	8.31	
PHQ9	Elective	36	20.31	4.14	0.533
	Emergency	31	19.65	4.48	
GAD7	Elective	36	11.97	1.87	0.015
	Emergency	31	10.71	2.27	
BPISF	Elective	36	7.97	1.23	0.267
	Emergency	31	8.29	1.07	

## Discussion

The present study attempted to examine the complex correlation between pain following surgery and psychological symptoms. The results highlight a noteworthy relationship between the occurrence of postoperative pain and the presentation of psychological symptoms, including sleeplessness.

The patients in our study sample were relatively older, with a mean age of 61.53 years. It is reported that older adults have an increased vulnerability to the negative effects of pain and pain-related incidents [15]. This increased vulnerability can be attributed to various factors, such as age-related physiological changes, higher prevalence of chronic health conditions, reduced pain tolerance, and potential age-related cognitive decline. Understanding the impact of pain on older adults is crucial for providing appropriate pain management strategies and interventions that address the unique needs of this population.

The results of the correlation analysis between insomnia, depression, anxiety, and pain provided insights into the potential relationships among these variables. Notably, the observed correlations were weak and not statistically significant. This finding suggested that the associations between these variables may not be substantial or consistent within the studied sample. It is important to note that, despite our study's focus on the relationship between pain and age, the correlations we observed were weak and did not reach statistical significance. This means that the association between age and pain intensity or pain-related incidents was not strong enough to confidently conclude that age alone was a significant factor. There could be several explanations for these weak and non-significant correlations. Firstly, pain is a complex phenomenon influenced by various factors, such as individual differences in pain perception, underlying health conditions, psychological factors, and social support networks. Age alone may not fully account for the variation in pain experiences among individuals.

Regarding insomnia and mental health, the weak positive correlations between the ISI score and both the PHQ-9 and GAD-7 scores indicate that there may be some degree of association between these variables. The lack of statistical significance suggested that this association may have been due to chance or a number of alternative factors not accounted for in the analysis. There was a weak positive correlation between the ISI and BPISF, which implies a potential link between insomnia and pain, but again, the lack of statistical significance casts doubts on the strength and consistency of this relationship.

The absence of correlation could potentially be attributed to inadequate statistical power. With a small sample of approximately 67 participants, it becomes more challenging to detect significant relationships between variables, and any correlation that exists may not be apparent.

The lack of correlation may also arise due to the presence of distinct subgroups within the data. For example, the relationship between two variables may be different for different subsets of data (e.g., acute and chronic symptoms).

Based on the current body of evidence, it is reasonable to conclude that pain and insomnia are correlated [16]. The presence of shared neurotransmitters and molecular connections between depression and pain, combined with the high prevalence of depression in our study and its impact on pain perception, suggests that there may be implications for treating both conditions simultaneously. To achieve improved outcomes, it is crucial to adopt a paradigm that assesses and addresses depression and pain concurrently [17].

A bidirectional relationship exists between pain conditions and anxiety disorders, each aggravating the other. Postoperative pain is significantly predicted by age, anxiety, and the type of operation [18].

Understanding the underlying mechanisms and associations will aid in the development of integrative interventions addressing both physical pain and psychiatric symptoms that could mitigate the severity of each condition and improve overall patient outcomes.

While there is a limited body of research specifically focused on postoperative pain in Arab cultures, it is essential to recognize and comprehend the cultural factors that influence pain perception and treatment approaches within Arab groups. By gaining a deeper understanding of these cultural impacts, we can enhance our knowledge and approach to addressing postoperative pain in Arab populations [9].

Limitations of this study include the relatively older group of patients, including patients treated at only one hospital, albeit from various surgical departments. Future research should include a wider age range, with younger participants included and patients recruited from other hospitals. Future research should also explore more nuanced mechanisms underlying the relationship between pain and psychiatric symptoms and evaluate the efficacy of integrated interventions in larger, diverse patient populations.

Open surgeries may involve larger incisions and more tissue stress than laparoscopic procedures, which leads to greater levels of immediate postoperative discomfort [19]. Comparable numbers of individuals in our study underwent laparoscopic or open surgery. Individual differences in pain perception and psychological reactions must be taken into consideration, even though there may be a general trend that laparoscopic surgeries produce less immediate pain following surgery than conventional procedures [20].

## Conclusions

This study represents the first examination of psychological factors influencing acute pain and its progression in an Arab population. The findings of this study underscore the prevalence of severe pain among patients undergoing surgical procedures. There was a statistically significant relationship between postsurgical pain and insomnia, psychiatric symptoms, or illnesses, emphasizing the need for comprehensive, patient-centered care models that address both the physical and psychological aspects of postsurgical recovery.
